# Distribution, Source Apportionment, and Risk Assessment of Heavy Metals in Surface Waters from a Glacier Basin on the Southeastern Tibetan Plateau

**DOI:** 10.3390/toxics14080672

**Published:** 2026-07-29

**Authors:** Xinyu Wen, Rui Zhang, Qianli Hong, Hui Li, Yan Yao, Meixian Mo, Binbin Ren, Huawei Zhang

**Affiliations:** 1 College of Geography and Land Engineering, Yuxi Normal University, Yuxi 653100, China; wenxinyu@yxnu.edu.cn (X.W.); yaoyan@yxnu.edu.cn (Y.Y.); mmx@yxnu.edu.cn (M.M.); 2 Faculty of Geography, Yunnan Normal University, Kunming 650500, China; gtjdxl@outlook.com (R.Z.); 17387296853@163.com (Q.H.); 3 School of Earth Sciences, Yunnan University, Kunming 650504, China; 18187769129@163.com; 4 College of Tourism, Geography, History and Culture, Hulunbuir University, Hailaer 021008, China; www.rbb001@163.com; 5 Meili Snow Mountains Cryosphere Changes Observation and Research Station of Yunnan Province, Deqin 674500, China

**Keywords:** heavy metals, surface waters, potential risks, source identification, Meili Snow Mountains glacier basin

## Abstract

Heavy metals in surface waters pose significant risks to aquatic ecosystems and human health. In this study, a comprehensive analysis of multiple heavy metals (V, Cr, Mn, Co, Ni, Cu, Zn, As and Cd) was performed using extensive surface water samples from the Meili Snow Mountains glacier basin, southeastern Tibetan Plateau. Results showed that heavy metal concentrations were higher in glacier meltwater than in downstream rivers, with significant variability observed in both the glacier basin (0.00300–33.4 μg/L) and downstream rivers (0.00500–2.85 μg/L), attributable to local geological processes and regional atmospheric deposition of transported particulate pollutants. Elevated total heavy metal concentrations at Sinong River and Qunatong River were primarily driven by uneven distribution of specific metals, likely from both anthropogenic and natural sources. Principal component analysis extracted three components that grouped heavy metals into Mn–Co–Ni–Cd, Cu–Zn, and V–Cr associations, indicating mixed geogenic and anthropogenic sources, with long-range atmospheric transport from surrounding polluted regions being a notable contributor. Risk assessments indicated low ecological risks (ERI < 150) and safe non–carcinogenic risks (HI < 1), with children exhibiting higher susceptibility than adults and As being the primary contributor. Although the carcinogenic risks (TCR: 10^−6^–10^−4^) were acceptable, ingestion of As and Ni posed higher risks, especially in the Yubeng River basin for adults. These findings provide valuable insights for water resource management and health protection in the southeastern Tibetan Plateau.

## 1. Introduction

Globally distributed surface waters, though vital to humanity, are extensively polluted. Primary pollutants include organic compounds, heavy metals (HMs) and nutrients. Among these, HMs are of particular concern due to their intrinsic biological toxicity, environmental persistence, and bioaccumulation potential. Some HMs (e.g., Cd, Cr, As, Pb) are highly toxic even at trace levels [1], whereas others (e.g., Zn, Cu, Mn) are essential for metabolism within safe thresholds [2]. Although HMs originate partly from natural processes like geological weathering, anthropogenic activities such as mining, smelting, manufacturing, sewage sludge disposal, and agrochemical application constitute the dominant source [3]. In recent decades, rapid industrial and agricultural expansion has rendered HM pollution both ubiquitous and escalating, posing an increasingly severe threat to ecological integrity and human health [4]. Given that healthy aquatic ecosystems underpin socioeconomic development, HM pollution has become a focal point in environmental research. Consequently, risk assessment methods and models developed by the United States Environmental Protection Agency (US EPA) and the National Institutes of Health have been widely adopted in scientific research to evaluate ecological and human health risks.

Atmospheric HMs undergo long-range transport through atmospheric circulation and are ultimately deposited onto glaciers in remote high-altitude and polar regions through dry and wet deposition processes. Consequently, glaciers serve as important reservoirs of these pollutants. The Tibetan Plateau (TP), the highest and largest plateau in Central and East Asia, covers about 1.22 million km^2^ with an average elevation exceeding 4000 m. Recognized as the “Third Pole” and the “Roof of the World”, it also holds the largest glacial ice volume in the mid-latitude and is often referred to as “Water Tower of Asia” [5]. Serving as the headwater region for major rivers such as the Yarlung Tsangpo, Yellow River, and Yangtze River, the TP supports about 40% of the global population [5,6]. Furthermore, it plays a vital role in maintaining regional ecological security, preserving biodiversity, and safeguarding water resources. Thus, the health of its aquatic ecosystems is crucial for sustaining downstream industrial and agricultural activities [1,7]. Importantly, its ecological influence extends to China and Southeast Asia [8].

Despite historically low population density, and minimal direct anthropogenic influence, the TP now faces increasing environmental pressures from anthropogenic activities, threatening its sustainable development and long-term ecological security. Located between China and India, the TP receives airborne HMs from these neighboring regions. Simultaneously, glacier melt driven by global warming is altering local hydrology and degrading water quality [6], while rapid agricultural and industrial development across the plateau has introduced HMs of varying concentrations into aquatic ecosystems [8,9]. Additionally, demographic and socioeconomic changes are raising the demand for high-quality water throughout the region. These factors underscore the essential need for a comprehensive risk assessment of surface waters in the TP′s glacial basins.

Although extensive research has investigated HMs in aquatic ecosystems across the TP from major river basins to local waters [10,11,12,13], the anthropogenically influenced southeastern TP remains a critical gap in understanding regional-scale water quality. To address this, this study conducted extensive sampling of river water and glacier meltwater across six river basins in the Meili Snow Mountains region. It aimed to (1) investigate the regional-scale HM concentrations, (2) identify their potential sources, and (3) assess the associated ecological and human health risks. By doing so, this study not only addresses local drinking water safety concerns but also provides a scientific basis for regional water pollution prevention and management strategies.

## 2. Sampling and Methods

### 2.1. Sample Collection

**Figure 1:**
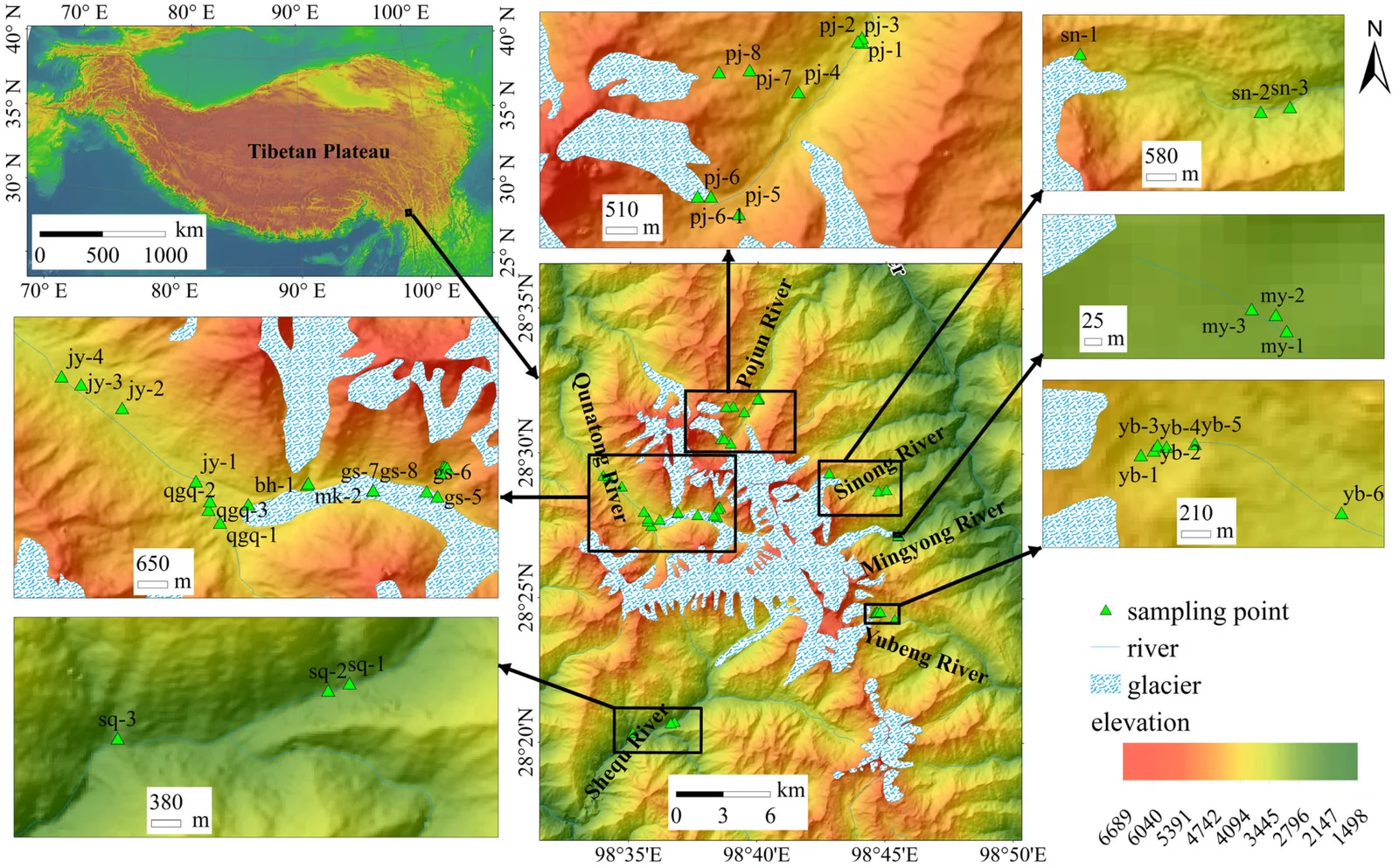
Map showing the sampling points and surrounding areas of the six river basins in the Meili Snow Mountains.

The study region is located within the glacial basins of the Meili Snow Mountains in southeastern TP. Field sampling was conducted in July 2023, during which a total of 42 water samples were collected. These comprised 31 glacier meltwater samples from the glacial basin and 11 river water samples from the downstream reaches of proglacial rivers. Sampling locations were grouped into six basins: the Qunatong River (QNTR), Shequ River (SQR), Yubeng River (YBR), Mingyong River (MYR), Sinong River (SNR), and Pojun River (PJR) (Figure 1). For the purpose of enhanced data analysis, the total of 42 water samples were categorized into 21 groups based on their source glacier and water type. Specifically, 13 groups comprised glacier meltwater samples, while the remaining 8 groups consisted of river water samples (Table S1). To prevent pollution and ensure analytical accuracy, clean polypropylene suits and gloves were worn during sampling, and an acid-cleaned stainless steel shovel was used for collection. In the field, water samples were filtered using 0.45 μm glass-fiber filters (Whatman International Ltd., Maidstone, England), and stored in acid-washed low-density polyethylene bottles (Thermo Scientific, Waltham, MA, USA) for HM analysis. All samples were kept in the dark at 4 °C until further processing.

### 2.2. Laboratory Analysis

All water samples were transferred to acid-washed polyethylene tubes for laboratory analysis. Each sample was acidified with 200 μL ultrapure nitric acid and adjusted to a final volume of 10 mL, and then stored at 4 °C in the dark prior to analysis. HM concentrations were determined using inductively coupled plasma–mass spectrometry (ICP-MS) (PerkinElmer Inc., Waltham, MA, USA) at the Institute for Ecological Research and Pollution Control of Plateau Lakes, Yunnan University. A total of 9 HMs (V, Cr, Mn, Co, Ni, Cu, Zn, As and Cd) were measured for subsequent environmental assessment.

To ensure data reliability, standard reference materials, method blanks, duplicate samples, and matrix-spiked samples were incorporated throughout the analytical process. Each batch of 20 samples included 1 reagent blank, 1 duplicate, 2 reference standard samples, and 1 matrix-spiked sample, yielding 15 valid data points. Precision and accuracy were verified using national certified reference materials (GSB 04-1767-2004), with differences between certified and measured values within 10% and parallel sample deviations below 5.0%. All elemental recovery rates fell between 91.8% and 110.6%.

### 2.3. Ecological Risk Assessment

The ecological risk index (ERI) provides a comprehensive evaluation of the overall ecological risks from HMs in surface waters, integrating their concentrations, ecological effects, and toxicological characteristics [14,15]. It is calculated as follows:

(1) $$ERI=\sum RI=\sum T_{i}\frac{C_{i}}{B_{i}}$$

where *RI* denotes the potential ecological risk factor for HM *i*, and *T_i_* is its toxic response factor (Table S2 [16,17]); *C_i_* is the measured concentration, and *B_i_* is the corresponding background value (Table S2). The *ERI* is classified into four levels: low (*ERI* < 150), moderate (150 ≤ *ERI* < 300), considerable (300 ≤ *ERI* < 600), and high (*ERI* ≥ 600) [18].

### 2.4. Health Risk Assessment

Health risk assessment is a widely used method for evaluating the likelihood and severity of adverse health effects from exposure to HMs, primarily via water ingestion and dermal absorption [19,20]. In this study, we adopted the US EPA recommended health risk assessment framework [21]; accordingly, the average daily dose (ADD) from ingestion (*ADD_ingestion_*) and dermal absorption (*ADD_dermal_*) is calculated using Equations (2) and (3), respectively.

(2) $${ADD}_{ingestion}=\frac{C_{i}\times IR\times EF\times ED}{BW\times AT}$$

(3) $${ADD}_{dermal}=\frac{C_{i}\times SA\times EF\times ED\times ET\times K_{p}}{BW\times AT}\times 10^{-3}$$

In this study, both the non-carcinogenic risk (NCR) and carcinogenic risk (CR) were evaluated. The NCR was quantified using the hazard quotient (HQ). The hazard index (HI), defined as the sum of individual HQs, reflects the overall NCR posed by HM exposure. HQ and HI were calculated using Equations (4) and (5), respectively.

(4) $$HQ=\frac{ADD}{RfD}$$

(5) $$HI=\sum {HQ}_{ingestion}+{HQ}_{dermal}$$

where *RfD* is defined as the toxicity reference dose.

If the *HQ* or *HI* exceeds 1, adverse health effects are possible; conversely, values below 1 indicate no significant health risk.

The CR quantifies the lifetime cancer risk posed by carcinogenic HMs and is computed using Equation (6).

(6) CR=ADD×CSF

Total carcinogenic risk (TCR) is the sum of the individual CR values, providing an integrated indicator of the potential carcinogenic impact of HM pollution.

(7) $$TCR=\sum ({CR}_{ingestion}+{CR}_{dermal})$$

Here, the CSF is the cancer slope factor (kg·d/mg). In this study, the CR of As, Ni, Cr and Cd via ingestion was evaluated using CSF values of 1.5, 1.7, 0.501 and 0.63 kg·d/mg, respectively [10]. For dermal exposure to As, CSF = 3.66 kg·d/mg was applied [19]. Other parameters in Equations (3)–(5) are summarized in Tables S3 and S4 [21,22,23,24,25,26,27,28]. CR or TCR values lying between 1 × 10^−4^ and 1 × 10^−6^ are generally considered acceptable, whereas values exceeding 1 × 10^−4^ indicate an unacceptable lifetime carcinogenic risk.

### 2.5. Monte Carlo Simulation

Monte Carlo simulation (MCS), a cornerstone of probabilistic risk assessment, overcomes the limitations of single-point evaluations by incorporating distribution-based inputs for HM concentrations and exposure factors [29,30,31]. To obtain realistic estimates of health risks, we employed the OnVeMCS v1.1 software to perform Monte Carlo simulations [32], evaluating both carcinogenic and non-carcinogenic probabilistic risks for adults and children. A preliminary sensitivity analysis was first conducted to identify the input parameters with the greatest influence on the risk outputs. These most sensitive variables were then assigned to the outer loop of a two-dimensional Monte Carlo simulation, while all other input parameters were placed in the inner loop. This two-stage procedure allowed us to rigorously propagate parameter importance and uncertainty, yielding more robust risk characterizations.

### 2.6. Statistical Analysis

Descriptive statistics (max, min, mean, standard deviation (SD), and coefficient of variation (CV)) for HMs were calculated using Excel 2022. The CV was used to quantitatively evaluate the spatial heterogeneity of each metal. Principal component analysis (PCA) was performed in SPSS 25. Correlations among HMs were assessed using Pearson′s method, with statistical significance set at *p* < 0.05 [14]. PCA was applied to identify potential sources of HMs in water samples [15,33], with eigenvalues serving as the criterion for extracting principal components (PCs).

### 2.7. Aerosol and Air Mass Backward Trajectories

To better identify potential sources of HMs, this study combined aerosol data with backward trajectory analysis. The aerosol data were obtained from the Visible Infrared Imaging Radiometer Suite (VIIRS) onboard the NOAA-20 satellite, using monthly Level-3 Deep Blue aerosol products, which provide global gridded Aerosol Optical Thickness (AOT) measurements over land and ocean. The Deep Blue algorithm builds on heritage methods developed for SeaWiFS and MODIS sensors [34,35]. Backward trajectories were generated with the NOAA HYSPLIT (Hybrid Single-Particle Lagrangian Integrated Trajectory) model. Five-day trajectories were computed for the one-year period prior to sampling (July 2022 to June 2023) at an arrival height of 1000 m, and cluster analysis was conducted at three-month intervals to assess the origins of air masses reaching the study area.

## 3. Results and Discussion

### 3.1. General Characteristics and Spatial Distribution

#### 3.1.1. Concentration Levels

Detailed statistical data are presented in Table 1. The distribution patterns of HMs differed markedly between the two water types. In glacier meltwater, the mean concentration followed the order: Mn (1.96 μg/L) > Zn (1.32 μg/L) > Ni (0.333 μg/L) > Cu (0.241 μg/L) > As (0.228 μg/L) > Cr (0.0790 μg/L) > Co (0.0370 μg/L) > V (0.0220 μg/L) > Cd (0.0130 μg/L). In river water, the order was: Zn (0.923 μg/L) > As (0.289 μg/L) > Mn (0.178 μg/L) > Ni (0.175 μg/L) > Cu (0.161 μg/L) > Cr (0.0800 μg/L) > V (0.0390 μg/L) > Cd (0.0120 μg/L) > Co (0.00900 μg/L). Furthermore, the CV analysis revealed that within the glacial basin, all HMs except Cd (CV = 41%) exhibited significant spatial heterogeneity (CV > 50%). Meanwhile in downstream rivers, similarly high variation was confined to V (51%), Zn (78%), and As (86%). This pattern implies that the two environments are affected by different point and non-point pollution sources [36], with the glacial basin experiencing more pronounced anthropogenic impacts [37].

#### 3.1.2. Comparative Abundance and Spatial Heterogeneity Between Glacial Meltwater and River Water

**Figure 2:**
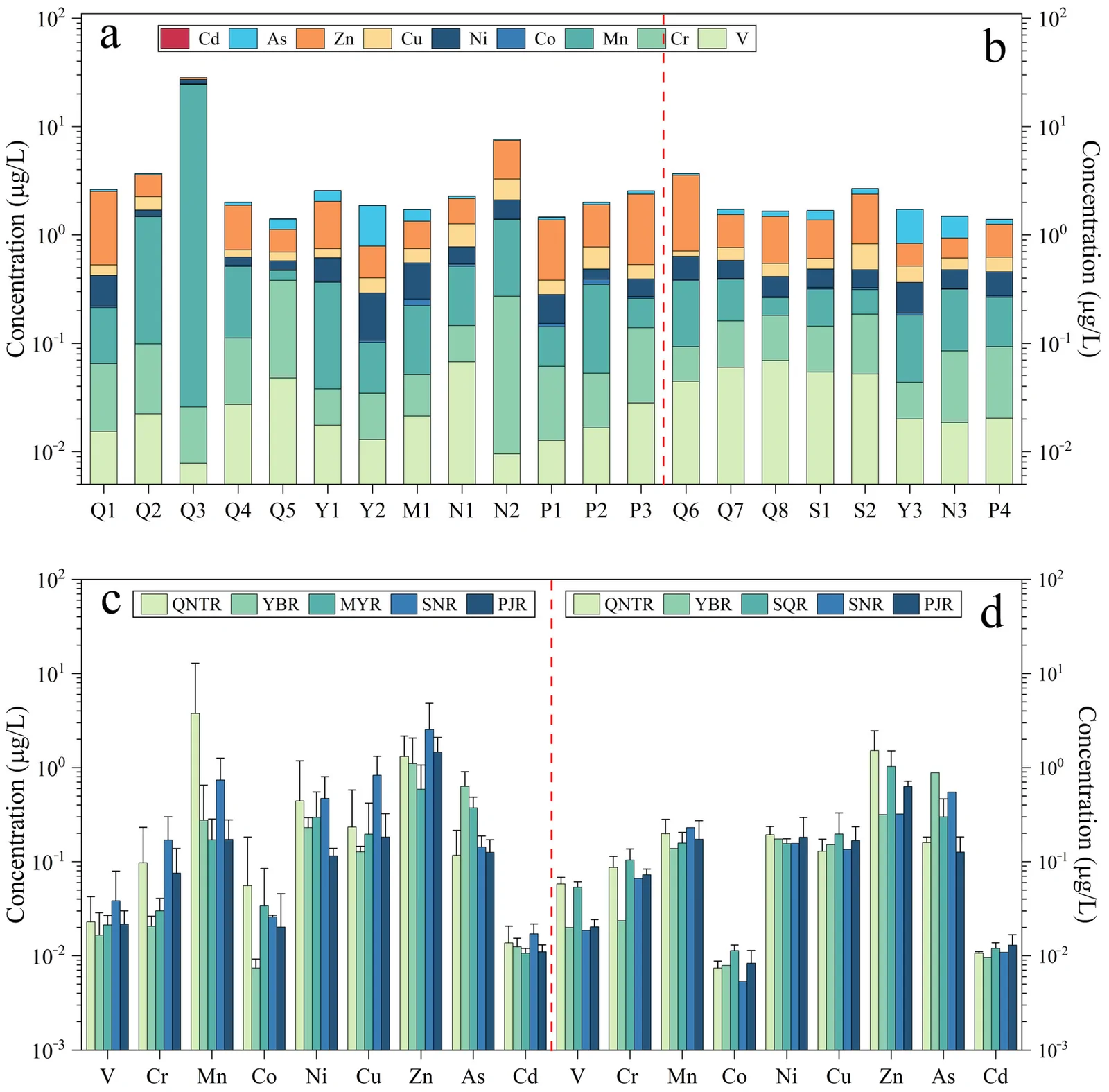
HM concentration of different sampling points, and the mean concentrations of different river basins ((**a**,**c**): glacier meltwater; (**b**,**d**): river water. Data on the bars and error bars are derived from the mean values and standard deviations of each basin).

Significant differences were observed in the spatial distribution and concentration ranges of HMs between the two water types. The total concentration of all HMs (∑HMs) was generally lower in downstream rivers than in glacial basins (Figure 2a,b). This is typically attributed to HMs derived from local geology (e.g., bedrock and aeolian dust) and regional atmospheric deposition of transported particulate pollutants in remote glacial basins [38,39]. Across the investigated basins, the mean ΣHMs in glacier meltwater was higher than in river water for most basins (SNR: 4.96 vs. 1.49 μg/L; QNTR: 6.05 vs. 2.36 μg/L; PJR: 2.19 vs. 1.39 μg/L; YBR: 2.43 vs. 1.72 μg/L). This pattern likely results from distinct glacier characteristics (e.g., intensive cryogenic weathering-pedogenesis, aeolian dust input, and regional atmospheric deposition), and local environmental conditions [40,41]. The spatial distribution of ΣHMs further illustrates this variability: within the glacier basin, concentrations ranged from a high of 28.3 μg/L at Q3 to a low of 1.41 μg/L at Q5; in downstream rivers, they varied from 3.69 μg/L at Q6 to 1.39 μg/L at P4. Notably, the Sinong and Gongsen Glaciers are situated in relatively closer proximity to local settlements compared to the other sampling sites. This geographical setting may render them more susceptible to potential anthropogenic influences, as they could receive enhanced atmospheric deposition and surface dust inputs from adjacent inhabited areas. It is therefore plausible that, if these influxes carry elevated levels of trace elements, such pollutants may be retained and progressively concentrated within cryoconite holes, ultimately contributing to the higher HM concentrations observed in the meltwater from these two glaciers.

We acknowledge, however, that this interpretation remains a tentative hypothesis rather than a directly validated mechanism, as the present study does not include independent measurements of cryoconite composition, local dust geochemistry, settlement density, tourism activity, or local emission inventories. While the spatial pattern—higher HM levels at these two proximal sites—is consistent with an anthropogenic influence, direct confirmation would require targeted sampling of cryoconite material, surface dust, and local source apportionment analyses. We have therefore framed this discussion as a plausible explanation for the observed spatial heterogeneity, while recognizing that alternative natural factors (e.g., differences in bedrock mineralogy or glacial hydrological regimes) cannot be entirely excluded and warrant future investigation.

The spatial variability of individual HMs was more pronounced in glacier meltwater than in river water within the same basin (Figure 2c,d). Both water types in the QNTR basin exhibited considerable spatial heterogeneity, as indicated by higher standard deviations compared to other basins. Overall, most HMs showed significant spatial variation in glacier meltwater (except Cd), whereas in river water, only V, Cr, Zn, and As displayed notable variability. In glacier meltwater, elevated concentrations of Mn and Co were observed in QNTR, while SNR showed higher levels of V, Cr, Cu, Zn, and Cd. YBR was characterized by having the highest Zn concentration (1.11 μg/L) and the highest As concentration (0.632 μg/L) alongside the lowest levels of V, Cr, Co, and Cu. In river water, QNTR exhibited higher concentrations of V, Ni, and Zn, while SQR had elevated Cr, Co, and Cu. Mirroring the meltwater trend, YBR in river water recorded the highest As concentration (0.881 μg/L) and the lowest Cr, Mn, and Cd levels.

#### 3.1.3. Dominant Contributions of Different HMs and Regional Characteristics

The elevated concentrations of Ni, Zn, Cu, and Cr across the investigated basins are likely influenced by anthropogenic emissions. Furthermore, the differing spatial peaks of these metals between water types suggest they may originate from distinct anthropogenic or natural sources. The elevated mean ΣHMs in surface waters are primarily driven by the uneven contribution of specific HMs, which may originate from different anthropogenic activities or natural sources [1,42]. For instance, in glacier meltwater, ∑HMs were largely driven by Mn in QNTR, and by Zn in the other four basins. In river water, however, As dominated in YBR and SNR, while Zn was the major contributor in the remaining three basins. The consistently high As concentrations in both glacier and river water from YBR suggest substantial occurrence of As in this basin. The observed downstream enrichment of As, despite recharge from glacier meltwater and precipitation during sampling, underscores the critical role of atmospheric wet deposition and glacier meltwater inputs. Overall, the atmospheric deposition of HM pollutants was generally higher in QNTR and SNR compared to the other basins.

**Figure 3:**
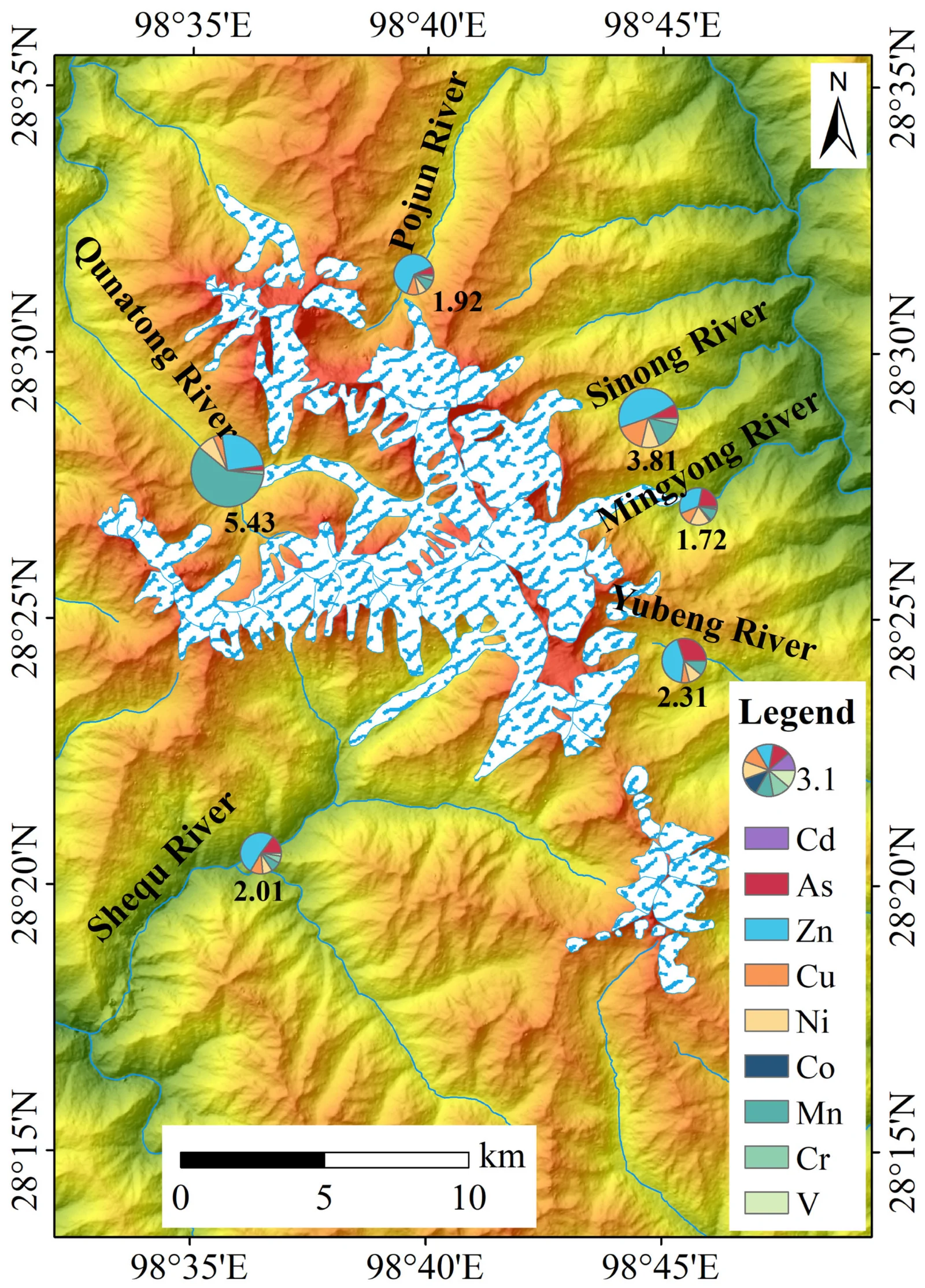
The mean concentration of HMs in water bodies of each river basin.

Among the river basins in the Meili Snow Mountain region (Figure 3), the Qunatong River Basin exhibits the highest total concentration of HMs in water (5.43 μg/L), while the Mingyong River Basin shows the lowest (1.72 μg/L). Except for the Qunatong River Basin, where Mn is predominant (3.16 μg/L), Zn is the dominant HM in all other basins, accounting for 34.2–61.7% of the total. Concentrations of Cd, Co, Cr, and V remain low across all basins.

#### 3.1.4. Comparative Analysis with the TP and Surrounding Regions

**Figure 4:**
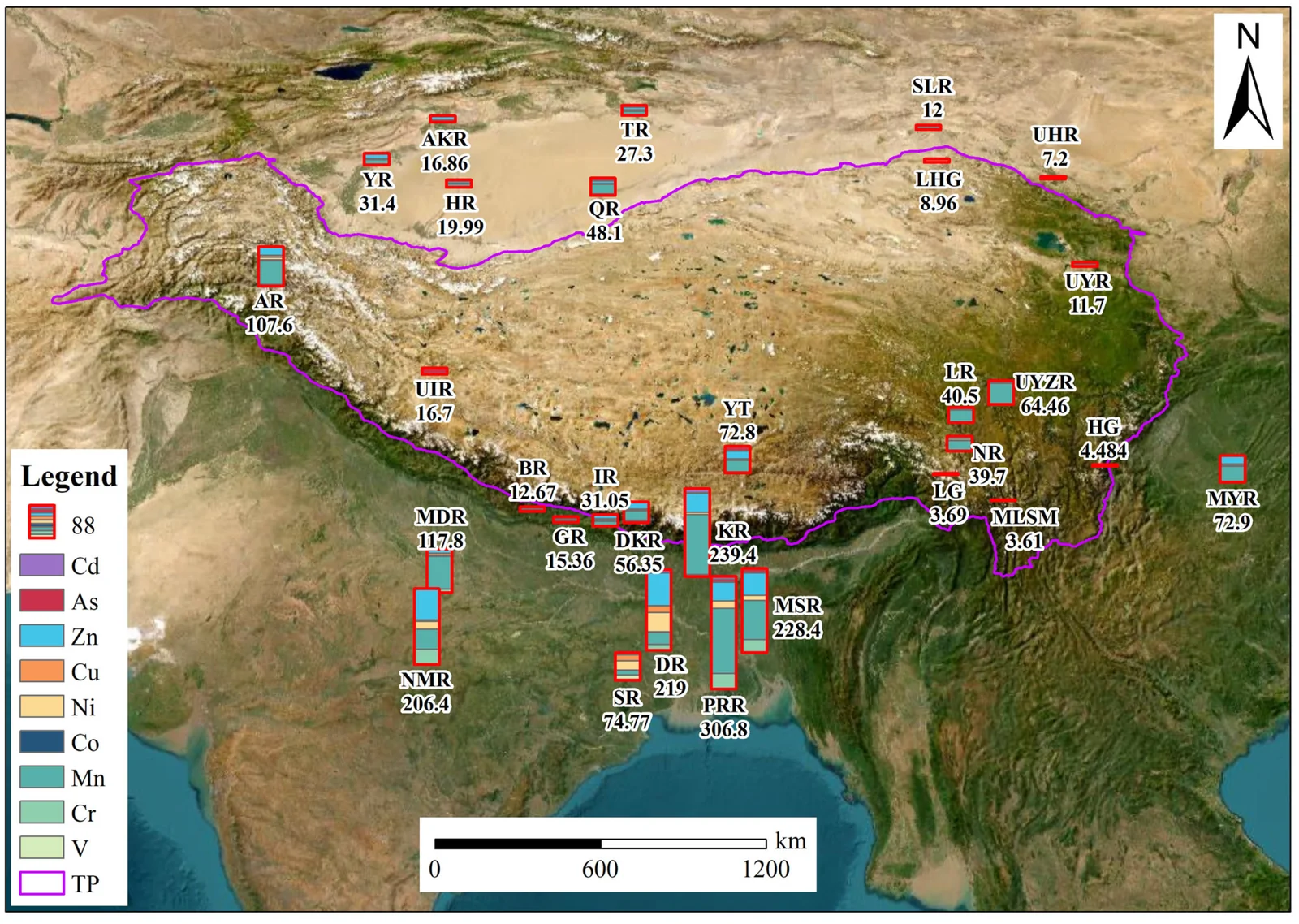
Distribution of mean HMs (μg/L) in water samples from various basin areas surrounding TP. AR represents Astore River [50], NMR represents Narmada River [43], BR represents Badigad River [47], DKR represents Dudh Koshi River, IR represents Indrawati River, GR represents Gandaki River [48], MDR represents Mandakini River [44], KR represents Karatoya River, PRR represents Pasur and Rupsha River, MSR represents Meghna and Shitalakshya River [19], DR represents Damodar River [45], SR represents Subarnarekha River [46], NR represents Nu River, LR represents Lancang River, UYZR represents the upper reaches of the Yangtze River [6], MYR represents the middle reaches of the Yangtze River [49], UIR represents the upper reaches of the Indus River, UYR represents the upper reaches of the Yellow River, UHR represents the upper reaches of the Hei River, SLR represents Shule River [7], YT represents Yarlung Tsangpo [51], LHG represents Laohugou Glacier [53], TR represents Tarim River, QR represents Qarqan River, HR represents Hotan River, YR represents Yarkant River, AKR represents Aksu River [52], LG represents Laigu Glacier, HG represents Hailuogou Glacier [1], and MLSM represents Meili Snow Mountains (this study).

Spatial heterogeneity in the mean concentrations of HMs (including As, Cd, Co, Cr, Cu, Mn, Ni, V, and Zn) is evident across river and glacial waters within and surrounding the TP (Figure 4). Elevated concentrations are observed in rivers from India and Bangladesh. Specifically, in Bangladesh, the highest concentration reaches 306.8 μg/L (range: 228.4–306.8 μg/L) in the Pasur and Rupsha River (PRR) basin [19]; in India, the highest concentration is 219 μg/L (range: 74.77–219 μg/L) in the Damodar River (DR) basin [43,44,45,46]. Rivers originating from the Himalayas in Nepal show comparatively lower concentrations (12.67–56.35 μg/L), with the Dudh Koshi River (DKR) being the highest [47,48].

Among the major rivers sourcing from the eastern and central TP (Yangtze, Yellow, Lancang, and Nujiang), the Yangtze River exhibits relatively higher concentrations, increasing from 64.46 μg/L in its upper reaches to 72.9 μg/L in the middle reaches [6,49]. The upper Yellow River shows the lowest concentration (11.7 μg/L) [7], while the Nujiang and Lancang Rivers measure 39.7 μg/L and 40.5 μg/L, respectively [6]. Within the Indus River Basin in the western plateau, concentrations vary significantly, with the upper reaches of the Indus River (UIR) at 16.7 μg/L and the Astore River (AR) reaching 107.6 μg/L [7,50]. The Yarlung Tsangpo River, originating from the northern slopes of the Himalayas, presents a moderate concentration of 72.8 μg/L [51].

In the northern TP, the Tarim River and its major tributaries (e.g., Qarqan, Hotan, Yarkant, and Aksu Rivers) exhibit higher HM concentrations (16.86–48.1 μg/L) compared to the Shule River (12 μg/L) and Heihe River (7.2 μg/L) [7,52].

In contrast, glacier meltwater samples (e.g., from Laigu, Hailuogou, Laohugou, and the Meili Snow Mountains in this study) consistently demonstrate low HM concentrations, ranging from 3.61 to 8.96 μg/L [1,53]. Across the studied water bodies, Mn and Zn are identified as the predominant contributors to the total HM content.

### 3.2. Source Identification of HMs

#### 3.2.1. Source Apportionment Based on Principal Component Analysis (PCA)

PCA was further employed as an exploratory tool to examine the grouping patterns of HMs and to gain preliminary insights into their potential sources. Notably, PCA was performed directly on the 42 individual samples to preserve statistical degrees of freedom. Prior to extraction, the Kaiser–Meyer–Olkin (KMO) measure of sampling adequacy was 0.694, and Bartlett’s test of sphericity was significant (*p* < 0.001), confirming that the dataset was acceptable for factor analysis. All data were standardized to z-scores to eliminate scale effects, and varimax rotation was applied to maximize the interpretability of component loadings.

Three principal components (PCs) with eigenvalues > 1 were retained, cumulatively explaining 76.6% of the total variance (Table 2). PC1, accounting for 42.57% of the variance, showed strong positive loadings for Mn, Co, Ni and Cd. PC2, explaining 18.77% of the variance, was heavily loaded with Cu and Zn. PC3, accounting for 15.26% of the variance, exhibited high loadings for V and Cr. Given the moderate sample-to-variable ratio (42 samples vs. 9 variables), these PCA results should be interpreted as exploratory indications of potential element associations rather than as a definitive quantitative source apportionment. The element groupings derived from the overall dataset are broadly consistent with known source signatures reported in the literature. For instance, Co, Mn, and Ni originate naturally from the weathering of parent materials and pedogenic processes, with additional anthropogenic inputs from industrial waste, coal combustion, and vehicle exhaust [28]. Cd, a highly toxic element, occurs naturally in regional soil parent material, but its anthropogenic inputs are primarily from heavy industrial activities, such as coal combustion, electroplating, battery manufacturing, non-ferrous metal smelting and mining [1,54]. Atmospheric Cr primarily derives from fossil fuel combustion, iron and steel plant emissions, waste incineration, and solid waste disposal [55]. V and Cr are often indicative of geogenic sources like bedrock weathering and mineral dust [56]. Cu and Zn are associated with industrial processes (e.g., metallurgy, petrochemical plants, and purification industries) [10,57], and are also common additives in livestock feed and organic fertilizers [58]. Notably, the Tibetan Plateau (TP) is rich in mineral resources, with Cu, Co, Ni, and Zn forming dominant deposits [59]. Elements like Ni, As and Cr are also frequently derived from fossil fuel combustion [40]. Therefore, while PCA suggests mixed origins with a predominant anthropogenic influence, quantitative source contributions cannot be conclusively determined from PCA alone. Future studies employing positive matrix factorization (PMF) combined with local emission inventory data are required to achieve a robust quantitative apportionment.

Previous studies confirm that HMs in the aquatic environment of the TP originate from multiple anthropogenic sources, including local industrial/agricultural activities, vehicle emissions, and long-range atmospheric transport [1,10,56,60,61]. The southeastern TP is surrounded by heavily polluted regions including India, Myanmar, and the Sichuan Basin; pollutants from these regions can be transported via long-range atmospheric circulation and subsequently deposited into various environmental media (soil, aerosols, snow, and ice) on the TP. Therefore, atmospheric aerosol deposition constitutes a non-negligible source of HMs in regional aquatic ecosystems.

#### 3.2.2. Regional Transport Influences Revealed by Aerosol Optical Depth and Backward Trajectories

**Figure 5:**
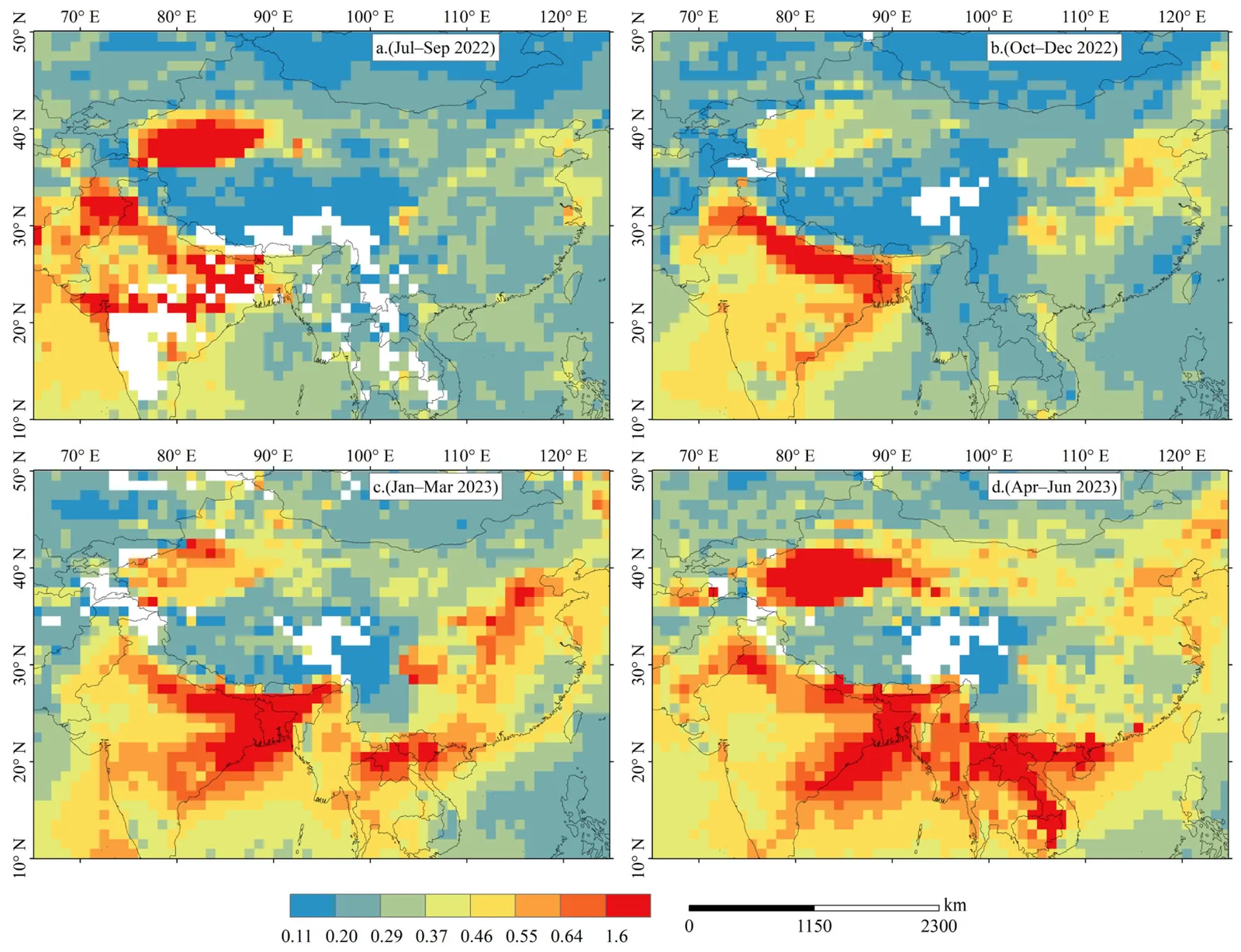
Aerosol Optical Thickness (AOT) from VIIRS/NOAA-20 during July 2022–June 2023 (https://ladsweb.modaps.eosdis.nasa.gov/ (accessed on 8 January 2026)).

Based on the spatial distribution of Aerosol Optical Thickness (AOT) (Figure 5), regions with elevated values during July–September 2022 were primarily located in the Tarim Basin, Pakistan, and northern India. From October to December 2022, the high-AOT area shifted to the southern foothills of the Himalayas. Between January and March 2023, high AOT concentrations were concentrated in northeastern India and Bangladesh. During April–June 2023, the extent of high AOT expanded, covering areas such as the Tarim Basin, northwestern India, Bangladesh, and Laos.

**Figure 6:**
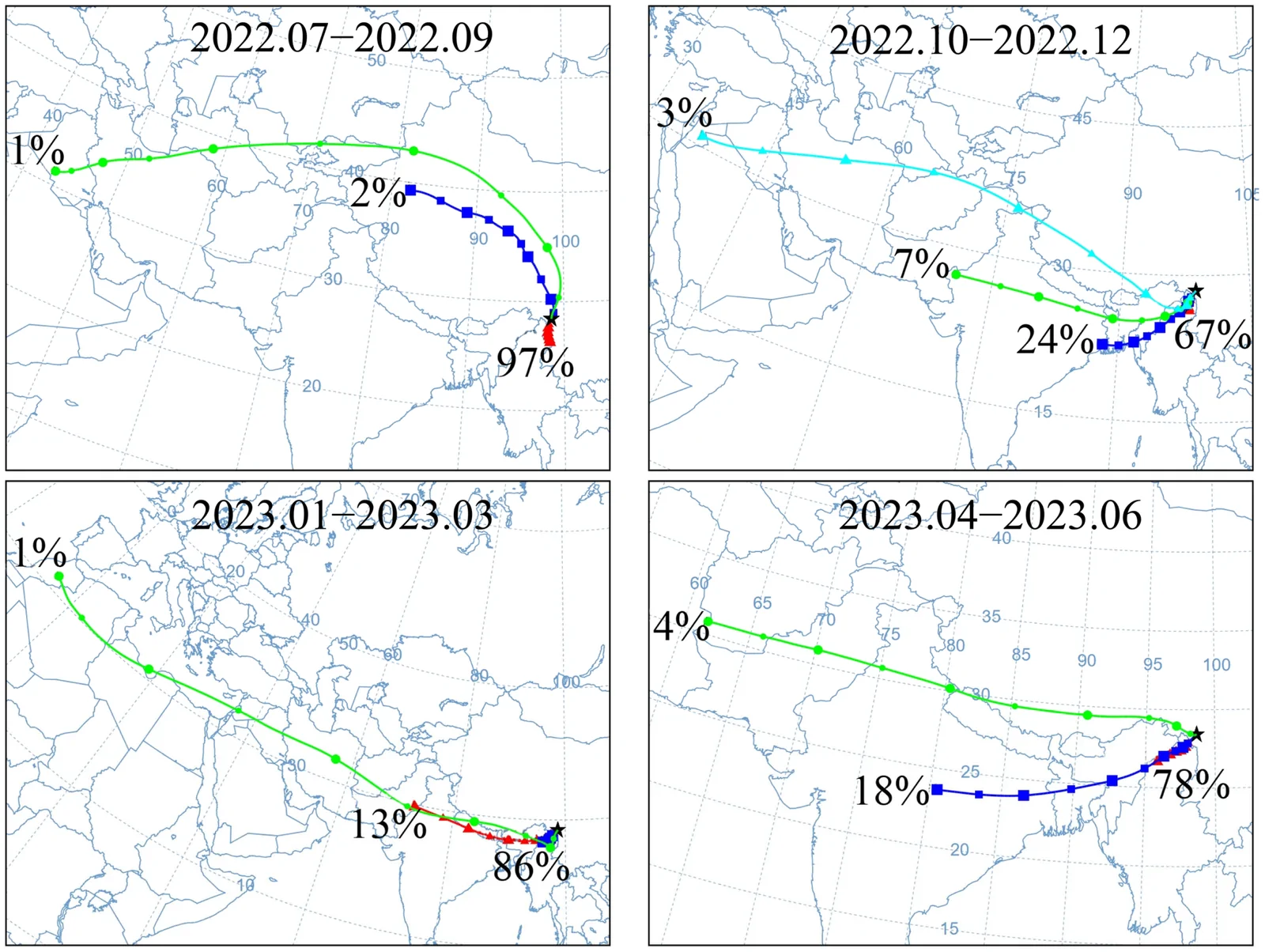
A cluster analysis of the 5-day backward trajectories of air masses in the Meili Snow Mountains from July 2022 to June 2023. The colored lines present different clusters.

Combined with backward trajectory analysis for the corresponding periods (Figure 6 [62]), these spatial–temporal patterns allow us to formulate a working hypothesis regarding the potential source regions of HMs in the study area. Specifically, the air masses arriving at the study site show possible pathways originating from the Tarim Basin during July–September 2022, and from India and Bangladesh during October 2022 to June 2023. However, it is important to emphasize that AOT reflects total aerosol column loading rather than chemical speciation, and HYSPLIT trajectories represent purely kinematic air-mass advection without carrying chemical fingerprints. Therefore, these results do not serve as direct evidence for the transport of specific metals (e.g., Cd, As, Ni, or Zn). Instead, they provide an indicative framework that points to potential external source areas. This hypothesis is further supported by previously published emission inventories and aerosol chemical composition studies in these regions [1,63,64,65], which consistently identify the Indo-Gangetic Plain and the Tarim Basin as major sources of dust-bound and anthropogenically derived trace elements. Nevertheless, direct verification through simultaneous aerosol chemical sampling, deposition flux measurements, or isotopic fingerprinting is required to conclusively confirm the actual contribution of these transported air masses to the local HM budget. With this caveat, our integrated analysis suggests that local and regional transport processes are the most plausible predominant contributors to HM levels in the study area, although this conclusion remains to be tested by future targeted chemical measurements.

### 3.3. Risk Assessment

#### 3.3.1. Ecological Risk Assessment

Certain HMs, such as Cd, Ni, and As, are highly toxic to aquatic organisms even at trace levels [66]. Others, such as Cr, Cu, Zn, and Mn, are essential micronutrients but become hazardous when their levels exceed safe thresholds [2]. Chronic exposure to elevated concentrations of HMs can impair aquatic ecosystem functions and pose risks to human health via ingestion or dermal contact.

**Figure 7:**
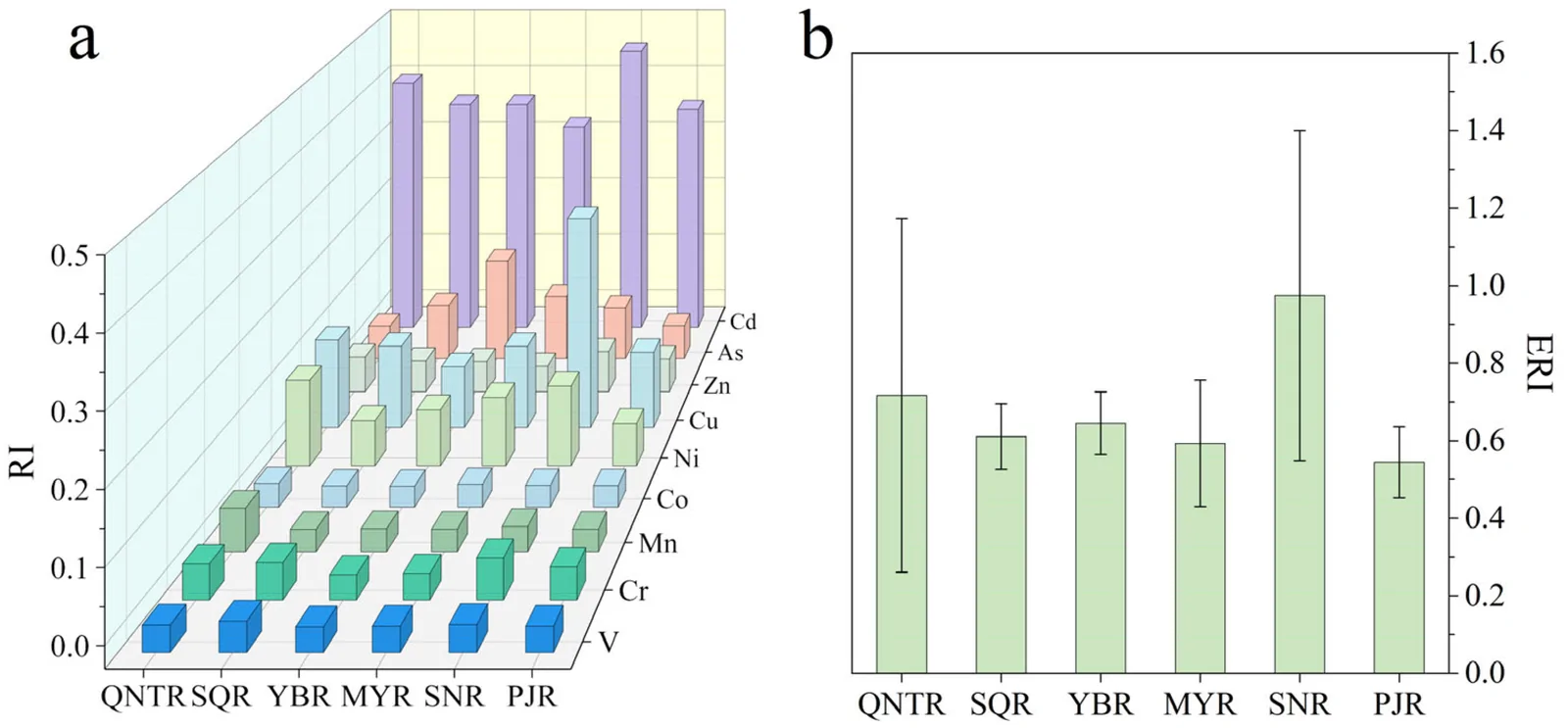
Mean RI (**a**) and ERI (**b**) values of HMs across different river basin.

In this study, concentrations of the nine analyzed HMs at all sampling points were well below the limits set by both the WHO and the Chinese drinking water standard [17,67], indicating that current water quality remains safe for domestic use with no identified risks. This finding aligns with previous reports confirming generally good water quality across the TP [11,68], attributable to minimal anthropogenic impact due to sparse population and limited industrial and agricultural activities. To further assess the potential impact of HMs on aquatic ecosystems in the southeastern TP, the RI and ERI were calculated for each basin (Figure 7). Spatial variability in RI was the smallest in PJR. Across all six basins, RI values varied significantly for all HMs except V and Co. The mean RI values followed the order: Cd > Cu > Ni > As > Zn > Cr > Mn > V > Co (range: 0.003–0.381). All ERI values were well below 150, collectively demonstrating a low overall ecological risk for surface waters across the Meili Snow Mountains glacier basin.

#### 3.3.2. Cancer Risk Assessment

**Figure 8:**
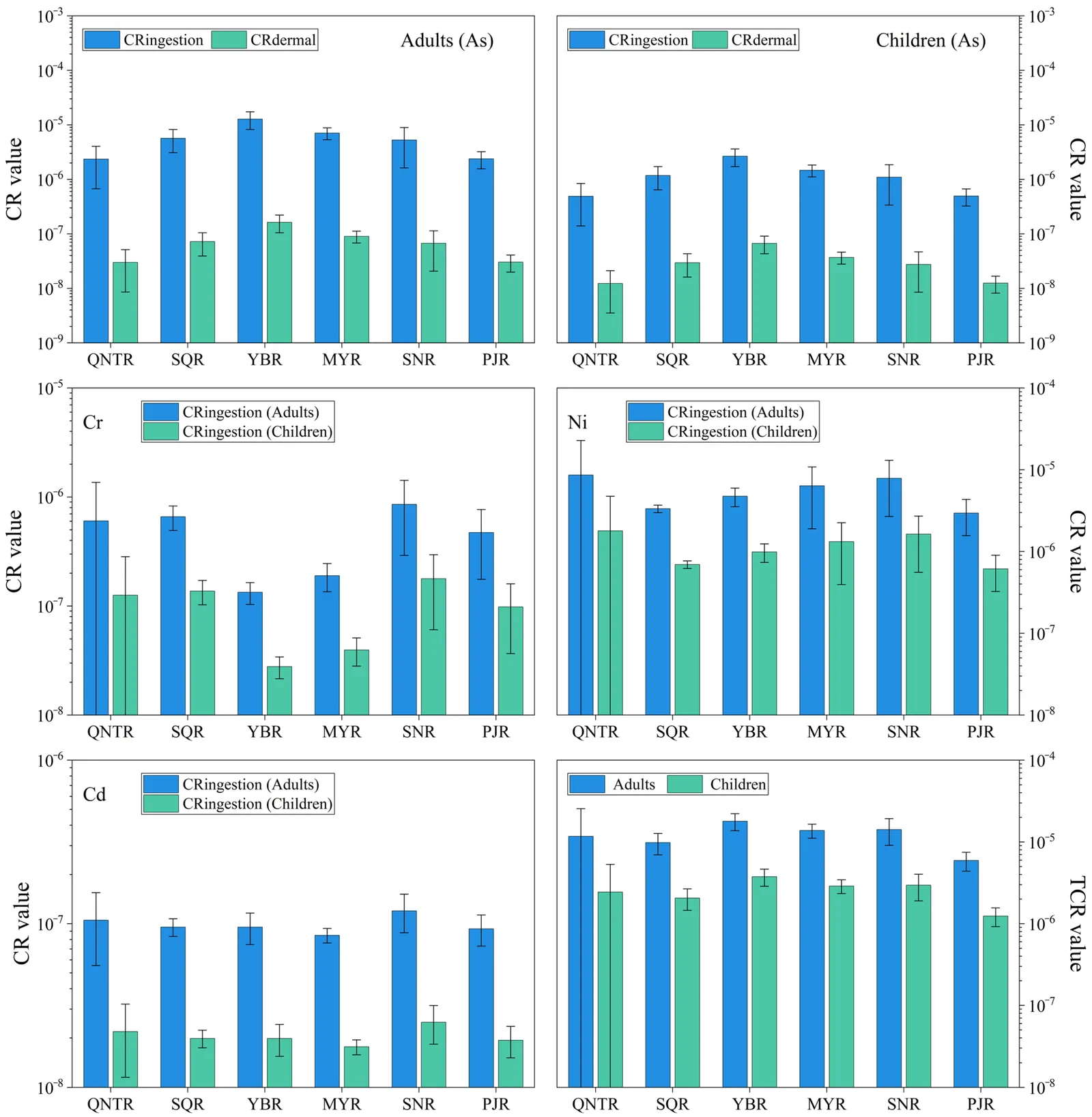
CR assessment of As, Cr, Ni and Cd for children and adults via ingestion and dermal exposure in different river basins.

As, Ni, Cr and Cd, identified as potential carcinogens [10], were the focus of CR assessment in this study. The calculated CR and TCR values are shown in Figure 8. The cancer risks via ingestion (CRingestion) and dermal (CRdermal) exposure exhibited distinct spatial patterns across the six basins for different metals. For As, both CRingestion and CRdermal followed the same descending order: YBR > MYR > SQR > SNR > PJR > QNTR. In contrast, Ni posed a relatively higher risk via ingestion, with a completely reversed spatial order: QNTR > SNR > MYR > YBR > SQR > PJR. Cr and Cd showed different patterns, with their CRingestion orders being SNR > SQR > QNTR > PJR > MYR > YBR and SNR > QNTR > SQR = YBR > PJR > MYR, respectively. For all metals and exposure routes, cancer risks for adults were consistently approximately one order of magnitude higher than those for children, likely due to longer exposure durations. The CRingestion values for Ni exceeded those for Cr and Cd by one order of magnitude for adults, and by one to two orders of magnitude for children. Notably, the CRingestion values of Ni were comparable to those of As for both age groups. Furthermore, the ingestion route contributed to significantly higher cancer risks for As than dermal exposure across all basins, a pattern consistent with findings from river basins in China and Bangladesh [69,70]. This underscores that As poses a significant risk to local residents primarily via drinking water ingestion. The mean CRingestion values for Ni were 6.26 × 10^−6^ for adults and 1.30 × 10^−6^ for children, while for As they were 4.63 × 10^−6^ and 9.63 × 10^−7^, respectively. These levels are considered unacceptable according to domestic standards [71], but fall within the acceptable range defined by USEPA [21]. Chronic high As intake is linked to serious diseases, including hypertension, diabetes, neuropathies, cardiovascular and cerebrovascular diseases, skin lesions, and cancers of the liver, lung, bladder, kidney, and skin [28,72]. Similarly, long-term Ni ingestion is associated with health effects ranging from acute gastrointestinal symptoms to nasal and lung cancers [4]. Therefore, As and Ni require special attention, necessitating measures to preserve healthy aquatic ecosystems.

The TCR values ranged from 2.63 × 10^−6^ to 5.16 × 10^−5^ (mean: 1.15 × 10^−5^) for adults and from 5.49 × 10^−7^ to 1.05 × 10^−5^ (mean: 2.41 × 10^−6^) for children. Most values fell between 10^−6^ and 10^−4^, indicating an acceptable risk level [21], with adults being more susceptible than children to combined HM exposure in the study area. The spatial distribution of mean TCR values was as follows: YBR (adults: 1.79 × 10^−5^, children: 3.76 × 10^−6^) > SNR (1.41 × 10^−5^, 2.96 × 10^−6^) > MYR (1.38 × 10^−5^, 2.89 × 10^−6^) > QNTR (1.17 × 10^−5^, 2.44 × 10^−6^) > SQR (9.83 × 10^−6^, 2.06 × 10^−6^) > PJR (5.93 × 10^−6^, 1.24 × 10^−6^). The elevated risks in YBR, particularly for both age groups, are likely linked to intensified human impacts from tourism development in Yubeng Village. The carcinogenic risk probability aligns with earlier findings from the TP [8,10]. Therefore, regular monitoring in investigated basins is recommended to prevent the accumulation of As, Ni, Cd, Cr, and other toxic metals that may pose serious health threats, especially to adults.

#### 3.3.3. Non-Cancer Risk Assessment

The NCR to human health from HMs in surface waters from six river basins was evaluated using the health risk assessment model recommended by the US EPA, with resulting HQingestion, HQdermal, and HI values presented in Table 3. Overall, the HI values were all below the threshold 1, suggesting no appreciable hazard from these basins. This is consistent with previous studies [11,67]. For both age groups, the HQingestion values remained below 1 (adults: 0.72 × 10^−4^ to 662.43 × 10^−4^, children: 0.75 × 10^−4^ to 688.93 × 10^−4^), implying that oral ingestion posed no significant adverse effects and a negligible NCR. The HQdermal values of HMs ranged from 0.01 × 10^−4^ to 5.07 × 10^−4^ for adults and 0.02 × 10^−4^ to 10.42 × 10^−4^ for children, similarly suggesting no health hazard via dermal contact. Notably, the children had consistently higher HQingestion, HQdermal, and HI values than adults (Table 3), reflecting their higher susceptibility under identical exposure conditions.

Among the studied HMs, As, Co, Mn, Cr and Cd were the major contributors to the elevated NCR for both children and adults. In particular, As showed the highest HQingestion and HI values, highlighting its potential detrimental impact on local residents’ health.

**Figure 9:**
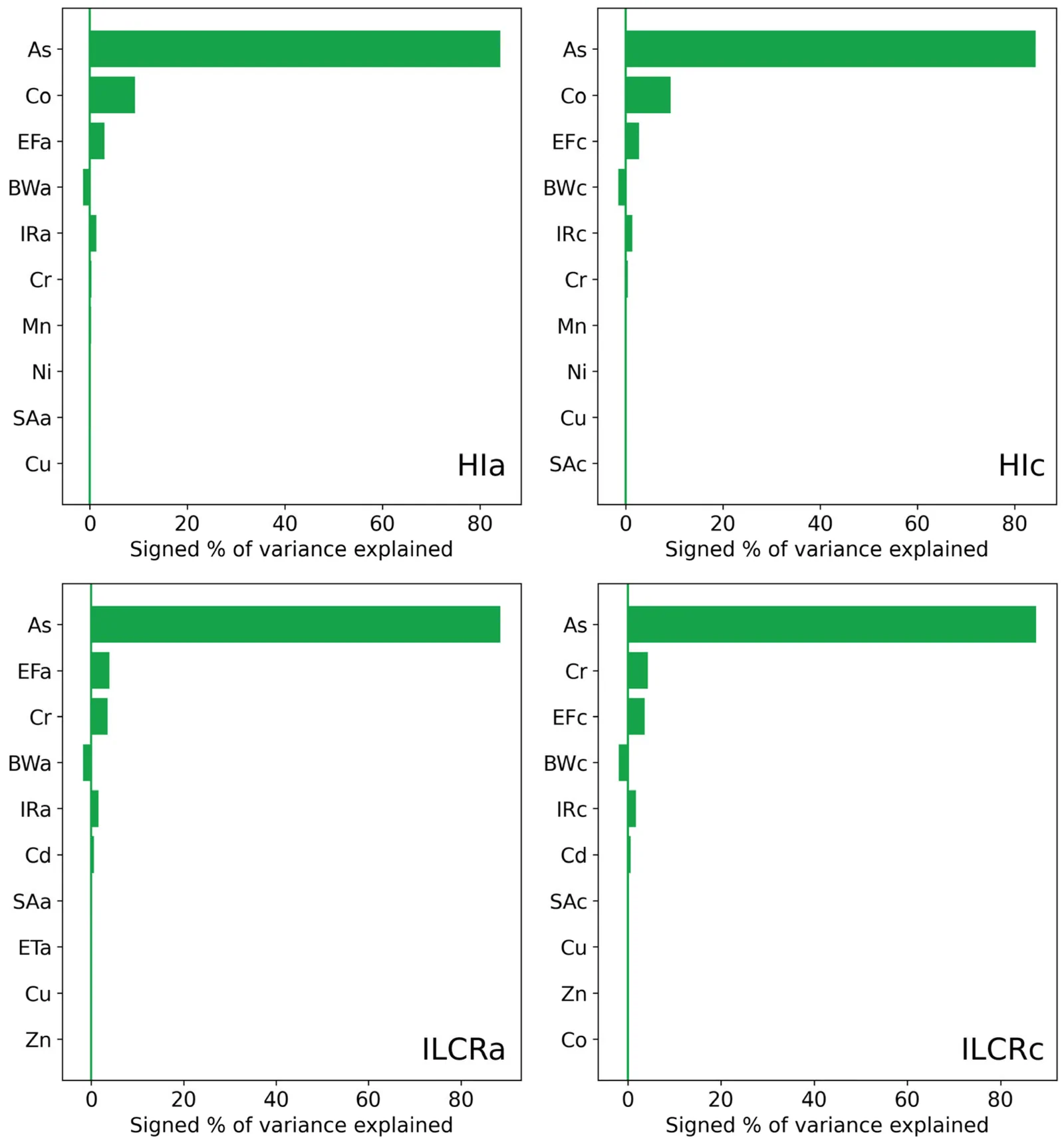
Sensitivity plot illustrating the effect of each input variable on non-carcinogenic risk for adults (HIa) and children (HIc), and total carcinogenic risk for adults (ILCRa) and children (ILCRc).

**Figure 10:**
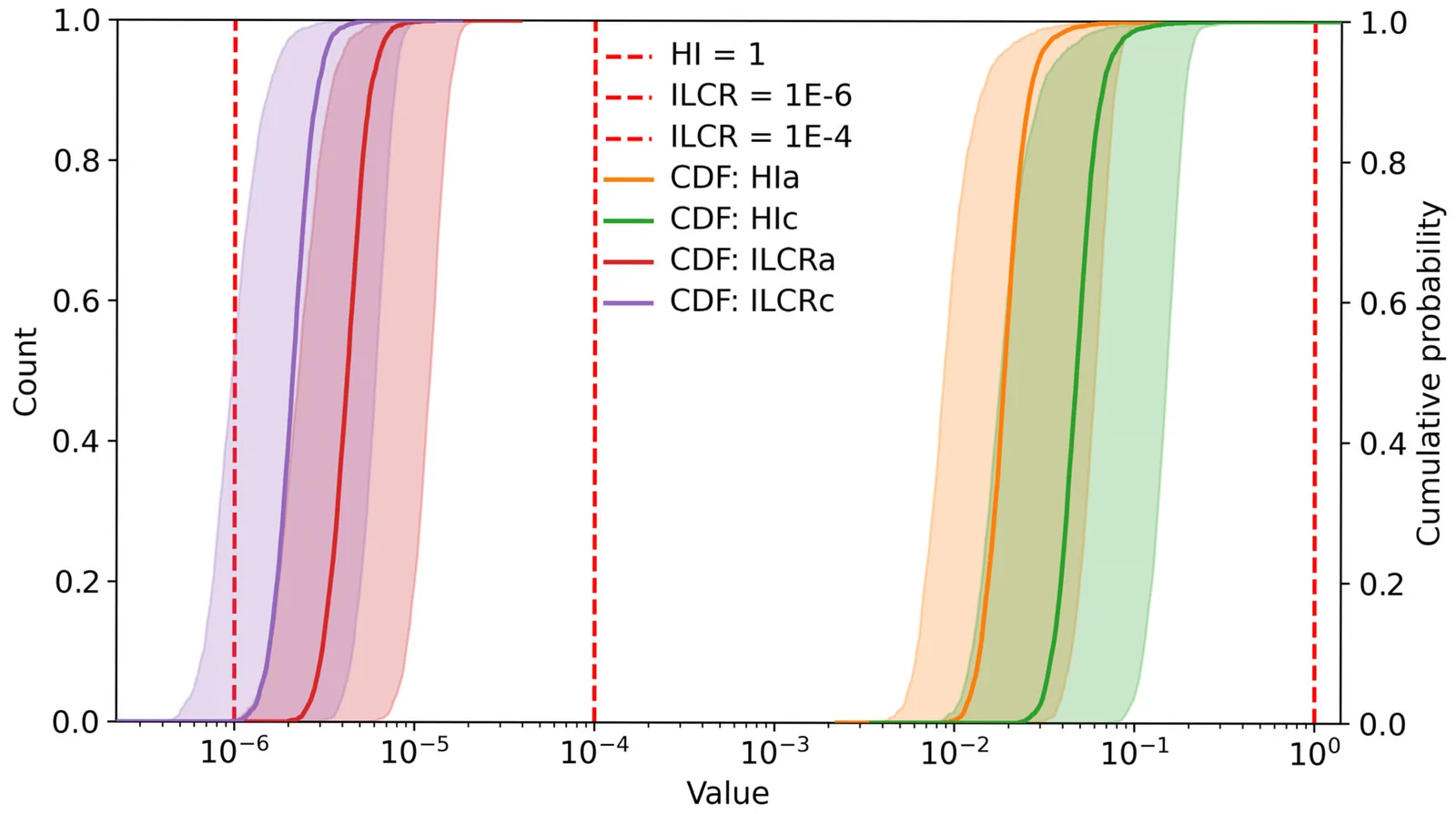
Cumulative distribution functions (CDFs) with confidence bands for the simulated risk metrics, illustrating model output distributions and uncertainty across Monte Carlo simulations.

Based on the sensitivity analysis of variables for non-carcinogenic and carcinogenic risks (Figure 9), As was identified as the most sensitive variable and was consequently designated as the outer loop parameter in the two-dimensional Monte Carlo simulation, with all remaining variables serving as inner loop parameters. This simulation generated the cumulative distribution functions (CDFs) with confidence bands for both non-carcinogenic risk (adults: HIa; children: HIc) and total carcinogenic risk (adults: ILCRa; children: ILCRc), as presented in Figure 10 (generated using the OnVeMCS v1.1 software).

In Figure 10, the vertical red dashed line at HI = 1 denotes the threshold for non-carcinogenic risk. All simulated HI values fall below unity, underscoring the advantage of probabilistic methods over deterministic point estimates, which might otherwise overestimate the risk. For carcinogenic risk, the dashed lines at 10^−6^ and 10^−4^ mark the USEPA’s lower and upper acceptable risk boundaries. For both ILCRa and ILCRc, most simulated values lie within this interval; however, the ILCRa curve is positioned consistently higher than its ILCRc counterpart under the simulated scenarios, indicating relatively greater cancer risk estimates for adults.

## 4. Conclusions

The TP, a unique region with a delicate ecological balance that supplies water resources to approximately 40% of the global population, plays a critical role in maintaining both ecological security and human health. In this study, we performed a comprehensive analysis of multiple HMs (V, Cr, Mn, Co, Ni, Cu, Zn, As and Cd) based on extensive surface water samples collected from the Meili Snow Mountains glacier basin in the southeastern TP. Results indicated that HM concentrations were significantly lower in downstream rivers than those in the glacier basin, with correspondingly lower pollution levels in river samples compared to glacier meltwater. Notably, concentrations varied significantly across six river basins regardless of water type. Principal component analysis (PCA) revealed three dominant components (cumulatively explaining 76.6% of the variance), which grouped heavy metals into Mn–Co–Ni–Cd, Cu–Zn, and V–Cr associations, indicating mixed geogenic and anthropogenic influences. The PCA-derived patterns are broadly consistent with known sources (e.g., industrial emissions, fossil fuel combustion, and parent material weathering), but the method alone does not allow quantitative source apportionment due to the moderate sample-to-variable ratio. Given the surrounding heavily polluted regions, long-range atmospheric transport represents a non-negligible contributor to heavy metal deposition in the Tibetan Plateau aquatic environment, and future application of positive matrix factorization (PMF) with local emission inventories is recommended for robust quantitative allocation. The combined AOT and trajectory analysis points to local and regional transport as the primary drivers of HMs, with periodic contributions from India and Bangladesh and lesser input from the Tarim Basin, though this conclusion awaits validation by targeted chemical measurements. The calculated ERI values indicated a low overall ecological risk associated with HMs. The NCR values (HI < 1) were within safe limits, with children exhibiting greater susceptibility than adults and As being the primary risk contributor. The TCR values of As, Ni, Cr, and Cd fell within the acceptable range (10^−6^–10^−4^); however ingestion of As and Ni posed elevated risks, particularly in the YBR basin where adults were more sensitive. Together, these results provide a critical baseline for guiding future pollution prevention, water resource management, and public health protection on the TP.

## Figures and Tables

**Table 1:** Summary statistics of trace element concentrations in glacial and river waters of the Tibetan Plateau.

Element	Glacier Meltwater					River Water				
	Mean	Max	Min	SD	CV(%)	Mean	Max	Min	SD	CV(%)
V	0.0220	0.0820	0.00600	0.017	76	0.0390	0.0690	0.0160	0.020	51
Cr	0.0790	0.566	0.0140	0.106	135	0.0800	0.134	0.0240	0.032	39
Mn	1.96	33.4	0.0520	6.479	331	0.178	0.282	0.0730	0.073	41
Co	0.0370	0.446	0.00300	0.090	244	0.00900	0.0120	0.00500	0.003	31
Ni	0.333	2.33	0.0680	0.531	160	0.175	0.313	0.108	0.059	34
Cu	0.241	1.43	0.0390	0.310	128	0.161	0.347	0.0730	0.076	48
Zn	1.32	4.16	0.229	0.941	72	0.923	2.85	0.316	0.722	78
As	0.228	1.08	0.0380	0.231	101	0.289	0.881	0.0920	0.247	86
Cd	0.0130	0.0310	0.00600	0.005	41	0.0120	0.0150	0.00900	0.002	19

**Table 2:** Variable rotated component matrix for HMs in glacier meltwater and river water.

Element	PC1	PC2	PC3
V	−0.164	−0.068	0.866
Cr	−0.048	0.124	0.883
Mn	0.964	−0.023	−0.097
Co	0.967	0.021	−0.114
Ni	0.951	0.111	−0.125
Cu	0.022	0.793	0.046
Zn	−0.040	0.851	−0.042
As	−0.189	−0.486	−0.043
Cd	0.903	0.216	−0.022
Eigenvalue	3.831	1.690	1.373
Variance (%)	42.57	18.77	15.26
Cumulative variance (%)	42.57	61.34	76.60

**Table 3:** NCR of HMs in surface water samples from six river basins, evaluated using HQ and HI (×10^−4^).

	QNTR		SQR		YBR		MYR		SNR		PJR	
	Adults	Children	Adults	Children	Adults	Children	Adults	Children	Adults	Children	Adults	Children
HQingestion												
V	1.21	1.26	2.25	2.34	0.72	0.75	0.90	0.93	1.34	1.39	0.90	0.93
Cr	9.39	9.76	10.25	10.66	2.08	2.16	2.95	3.07	13.30	13.83	7.33	7.63
Mn	38.85	40.41	1.94	2.02	3.11	3.23	2.09	2.18	6.98	7.26	2.12	2.20
Co	46.82	48.69	11.15	11.59	7.35	7.65	33.38	34.72	18.69	19.43	15.97	16.61
Ni	5.92	6.15	2.29	2.38	3.26	3.39	4.37	4.54	5.39	5.61	2.03	2.11
Cu	1.59	1.66	1.45	1.51	0.97	1.01	1.45	1.51	4.42	4.59	1.31	1.36
Zn	1.32	1.38	1.01	1.05	0.96	1.00	0.58	0.60	1.76	1.84	1.17	1.21
As	121.97	126.85	293.35	305.08	662.43	688.93	366.30	380.95	273.24	284.17	123.50	128.44
Cd	7.78	8.10	7.06	7.34	7.06	7.34	6.28	6.54	8.88	9.23	6.89	7.16
HQdermal												
V	0.63	1.30	1.18	2.42	0.38	0.78	0.47	0.96	0.70	1.44	0.47	0.96
Cr	1.96	4.03	2.14	4.40	0.43	0.89	0.62	1.27	2.78	5.71	1.53	3.15
Mn	5.07	10.42	0.25	0.52	0.41	0.83	0.27	0.56	0.91	1.87	0.28	0.57
Co	0.10	0.20	0.02	0.05	0.02	0.03	0.07	0.14	0.04	0.08	0.03	0.07
Ni	0.15	0.32	0.06	0.12	0.09	0.17	0.11	0.23	0.14	0.29	0.05	0.11
Cu	0.04	0.09	0.04	0.08	0.03	0.05	0.04	0.08	0.12	0.24	0.03	0.07
Zn	0.02	0.04	0.02	0.03	0.01	0.03	0.01	0.02	0.03	0.06	0.02	0.04
As	0.67	1.38	1.61	3.31	3.64	7.48	2.01	4.14	1.50	3.08	0.68	1.39
Cd	0.81	1.67	0.74	1.51	0.74	1.51	0.66	1.35	0.93	1.90	0.72	1.48
HI												
V	1.84	2.56	3.43	4.76	1.10	1.53	1.37	1.90	2.04	2.83	1.36	1.89
Cr	11.35	13.79	12.38	15.05	2.5	3.05	3.57	4.34	16.08	19.54	8.87	10.77
Mn	43.92	50.83	2.19	2.54	3.51	4.07	2.37	2.74	7.89	9.13	2.40	2.77
Co	46.91	48.89	11.17	11.64	7.37	7.68	33.45	34.86	18.72	19.51	16.00	16.67
Ni	6.07	6.47	2.35	2.51	3.35	3.57	4.48	4.78	5.54	5.90	2.08	2.22
Cu	1.64	1.74	1.49	1.59	0.99	1.06	1.49	1.58	4.53	4.83	1.34	1.43
Zn	1.34	1.42	1.02	1.08	0.97	1.03	0.59	0.62	1.79	1.89	1.18	1.25
As	122.64	128.23	294.96	308.39	666.07	696.41	368.31	385.09	274.74	287.26	124.18	129.84
Cd	8.60	9.77	7.80	8.86	7.80	8.86	6.94	7.88	9.80	11.13	7.61	8.64

## Data Availability

The original contributions presented in this study are included in the article/Supplementary Materials. Further inquiries can be directed towards the corresponding authors.

## Supplementary Materials

The following supporting information can be downloaded at: https://www.mdpi.com/article/10.3390/toxics14080672/s1. Table S1: The types of water samples and the correspondence between new and old IDs; Table S2: Parameters for indices’ calculation in the surface water; Table S3: Exposure and toxicological parameters used in health risk assessment; Table S4: Reference dose (RfD) and dermal permeability coefficient in samples (Kp) of HMs used for health risk assessment.

## Abbreviations

TP	Tibetan Plateau
QNTR	Qunatong River
SQR	Shequ River
YBR	Yubeng River
MYR	Mingyong River
SNR	Sinong River
PJR	Pojun River
ICP–MS	Inductively coupled plasma–mass spectrometry
ERI	Ecological risk index
NCR	Non-carcinogenic risk
US EPA	United States Environmental Protection Agency
TCR	Total carcinogenic risk
CSF	Cancer slope factor
CV	Coefficient of variation
SD	Standard deviation
PCA	Principal component analysis
PCs	Principal components
VIIRS	Visible Infrared Imaging Radiometer Suite
AOT	Aerosol Optical Thickness
HYSPLIT	Hybrid Single Particle Lagrangian Integrated Trajectory
HMs	Heavy metals
MCS	Monte Carlo simulation
ADD	average daily dose
HQ	hazard quotient
HI	hazard index
CDF	Cumulative distribution function
PMF	Positive matrix factorization
