# Supplementary material for: Distribution, Source Apportionment, and Risk Assessment of Heavy Metals in Surface Waters from a Glacier Basin on the Southeastern Tibetan Plateau
Source: Toxics. 2026 Jul 29;14(8):672. doi: 10.3390/toxics14080672 (PMC13517629; doi:10.3390/toxics14080672)
Supplement: Supplementary file 1 [file toxics-14-00672-s001.zip › toxics-4424507-supplementary.pdf]

Table S1 The types of water samples and the correspondence between new and old IDs

old ID	water type	new ID
gs-1, gs-2, gs-3, gs-4	glacier meltwater	Q1
gs-5, gs-6 ,gs-7, gs-8	glacier meltwater	Q2
mk-1, mk-2	glacier meltwater	Q3
qgq-1, qgq-2, qgq-3	glacier meltwater	Q4
jy-2, jy-3	glacier meltwater	Q5
yb-1, yb-2, yb-3, yb-4	glacier meltwater	Y1
yb-5	glacier meltwater	Y2
my-1, my-2, my-3	glacier meltwater	M1
sn-1	glacier meltwater	N1
sn-3	glacier meltwater	N2
pj-5	glacier meltwater	P1
pj-6, pj-6-1	glacier meltwater	P2
pj-4, pj-7, pj-8	glacier meltwater	P3
bh-1	river water	Q6
jy-1	river water	Q7
jy-4	river water	Q8
sq-1, sq-2	river water	S1
sq-3	river water	S2
yb-6	river water	Y3
sn-4	river water	S3
pj-1, pj-2, pj-3	river water	P4

Table S2 Parameters for indices calculation in surface waters

	Cu	Ni	As	Cd	Cr	Zn	Co	V	Mn
Ti	5	5	10	30	2	1	5	2	1
Bi (μg/L)	10[a]	20[b]	50[a]	1[a]	10[a]	50[a]	50[b]	10[b]	100[b]

[a]16; [b]17

Table S3 Exposure and toxicological parameters used in health risk assessment

Parameters	Meaning	Unit	Child	Adult	Reference
IR	Ingestion rate	L/day	0.64	2.0	22
EF	Exposure frequency	days/year	350	350	23
ED	Exposure duration	years	6	30	21
BW	Body weight	kg	20	65	24
AT	Average time for carcinogenic/non-carcinogenic effect	days	70×365/ED×365		25
SA	Exposed skin area	cm^2	6600	18000	
ET	Exposure time	h/day	1	0.58	
C_i	Concentration	μg/L			

Table S4 Reference dose (RfD) and dermal permeability coefficient in samples (Kp) of heavy metals used for health risk assessment [26-28].

	As	Cd	Cr	Cu	Zn	Mn	Ni	V	Co
RfDingestion (μg/(kg•day))	0.3	0.5	3	40	300	24	20	7	0.3
RfDdermal (μg/(kg•day))	0.285	0.025	0.075	8	60	0.96	0.8	0.07	0.3
Kp (cm/h)	1×10^{-3}	1×10^{-3}	1×10^{-3}	1×10^{-3}	6×10^{-4}	1×10^{-3}	2×10^{-4}	1×10^{-3}	4×10^{-4}
